# Mental Health Promotion and Stigma Reduction Among University Students Using the Reach, Efficacy, Adoption, Implementation, and Maintenance (RE-AIM) Framework: Protocol for a Mixed Methods Study

**DOI:** 10.2196/25592

**Published:** 2021-08-26

**Authors:** Kenneth Fung, Sheng-Li Cheng, Xuan Ning, Alan Tai-Wai Li, Jingxuan Zhang, Jenny Jing-Wen Liu, Carla T Hilario, Xiaojing Cheng, Miao Yu, Cun-Xian Jia, Jianguo Gao, Josephine Pui-Hing Wong

**Affiliations:** 1 Department of Psychiatry University of Toronto Toronto, ON Canada; 2 Department of Social Work School of Philosophy and Social Development Shandong University Jinan China; 3 Daphne Cockwell School of Nursing Ryerson University Toronto, ON Canada; 4 Regent Park Community Health Centre Toronto, ON Canada; 5 Shandong Mental Health Centre Jinan China; 6 University of Alberta Calgary, AB Canada; 7 Department of Epidemiology Shandong University Jinan China

**Keywords:** China, implementation science, intervention, mental health, stigma reduction, university students

## Abstract

**Background:**

Rapid urbanization, academic pressures, and developmental life transition stressors contribute to mental health stress for postsecondary students in China. Effective prevention, early identification, and timely intervention are challenged by stigma, a lack of mental health literacy, and inadequate mental health resources.

**Objective:**

Our implementation science (IS) research project is aimed at evaluating the use of an evidence-informed mental health promotion intervention named Acceptance and Commitment to Empowerment – Linking Youth and ‘Xin’ (hearts) (ACE-LYNX) to promote university student mental health in Jinan, China.

**Methods:**

We will engage and collaborate with Shandong Mental Health Center, the provincial mental health center, and six local universities in different regions of Jinan. The ACE-LYNX intervention aims to reduce social stigma against mental illness, enhance mental health literacy, and improve access to quality mental health care by increasing interdisciplinary collaboration and forming a mental health network. It is based on two evidence-based approaches, Acceptance and Commitment Therapy (ACT) and Group Empowerment Psychoeducation (GEP), and it will be delivered through online learning and in-person group training. The project will train 90 interdisciplinary professionals using the model. They will in turn train 15 professionals and 20 students at each university. The project will adopt the Reach, Efficacy, Adoption, Implementation, and Maintenance (RE-AIM) framework, which provides a structure to examine the process and outcomes of implementation using mixed methods comprising quantitative and qualitative approaches along five dimensions: reach, efficacy, adoption, implementation, and maintenance.

**Results:**

Over the course of the project, 720 champions will be directly trained. They will contribute to developing a formal and informal mental health network, strengthened by student-led mental health initiatives and professional-led initiatives to promote collaborative care and facilitated care pathways. We anticipate that our project will reach out to 11,000 to 18,000 students.

**Conclusions:**

This IS protocol will outline our unique intervention model and key steps to contextualize, implement, and evaluate community-based mental health intervention.

**International Registered Report Identifier (IRRID):**

PRR1-10.2196/25592

## Introduction

In China, rapid urbanization and mass internal migration to big cities have led to increasing concerns about stress and mental health risks for people living in big cities [1]. Based on a national survey from 2013 to 2015, there is an estimated lifetime prevalence of 16.6% for all mental disorders excluding dementia, with the prevalence of most disorders being higher than that observed in the 2002 survey [2]. Youths constitute the age group particularly vulnerable to the first onset of major mental illness. The prevalence rate of anxiety disorders and depression among children and youths in China were 24% and 16%, respectively, in 2014 [3].

For university students in China, the prevalence rate of various mental disorders is as high as 20% to 30% [4,5]. This is particularly alarming as Chinese students make up one in five of the world’s postsecondary student population [6]. Academic stress and performance requirements, high-pressure university majors or programs, minority status, family income, and ineffective coping were identified as factors contributing to university students’ stress and mental disorders, including anxiety, depression, suicidal ideation, alcoholism, and self-harm [5,7-10]. As there is increasing evidence suggesting an association between the duration of untreated illness and clinical outcomes, it is of critical importance to implement effective prevention, early identification, and timely intervention initiatives [11].

Given the significant mental health needs, researchers, health care providers, and policy makers in China have called for higher prioritization of mental health promotion, prevention, and care [2]. A number of studies in China have found that the general public across different levels of education have limited knowledge about mental disorders, and mental disorders are often associated with stigma and misattributed to personality weaknesses or social skill deficits [12]. Mental health literacy strategies address this problem by increasing the ability of the target population to recognize common mental illnesses and learn how to seek mental health information or help [13]. At the systemic level, another factor is the shortage of trained mental health professionals and the lack of adequate mental health resources to match the population’s growing needs [2]. Thus, strategies to promote mental health should address multiple barriers impeding help-seeking, including mental illness stigma, mental health literacy, and the capacity of service providers to respond to mental health needs [10,13].

Our implementation science (IS) research project named Acceptance and Commitment to Empowerment–Linking Youth and “Xin” (hearts) (ACE-LYNX) is a five-year Canada–China collaborative project aiming to promote the mental health and well-being of university students in Jinan, China. The project uses an evidence-informed multipronged approach to reduce stigma against mental illness, enhance mental health literacy, and improve access to quality mental health care by promoting interdisciplinary collaboration among service providers. The vision is to engage service providers and students to collaborate in developing formal and informal interdisciplinary mental health care networks to improve access to mental health services and peer support. As an IS project, it consists of two main phases: contextual analysis with service providers and students to inform the contextual adaptation of the ACE-LYNX intervention [14]; and the implementation phase of the ACE-LYNX intervention with service providers and university students, the focus of this protocol paper. Integrative knowledge translation (iKT) will be applied in all the stages of this study (paper under preparation) [15].

## Methods

### Project Location: Jinan, Shandong, China

Linking Hearts will take place in Jinan, a metropolis city with a population of over 7 million [1]. We will engage and collaborate with Shandong Mental Health Center, the provincial mental health center, and six local universities in different regions of Jinan, including Shandong University (63,185 students), University of Jinan (35,916 students), Shandong Jianzhu University (26,000 students), Shandong Youth University of Political Science (12,500 students), Shandong Normal University (36,456 students), and Shandong Women’s University (11,410 students).

### Project Intervention: The ACE-LYNX Intervention

**Figure 1:**
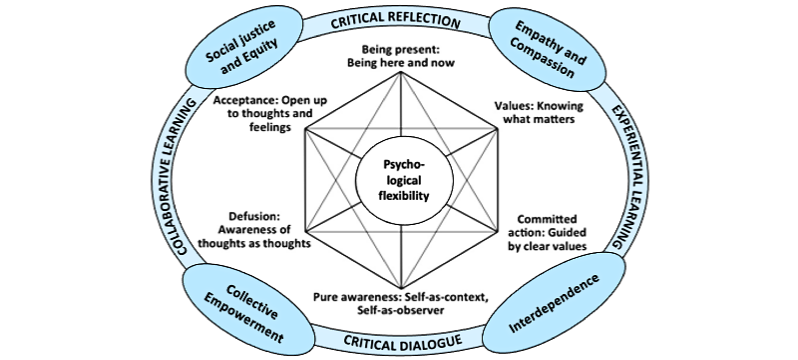
Acceptance and Commitment to Empowerment–Linking Youth and ‘Xin’ intervention presented as an integrated model of Acceptance and Commitment Therapy and Group Empowerment Psychotherapy.

ACE-LYNX is an integrated model of intervention based on two evidence-based approaches: Acceptance and Commitment Therapy (ACT) and Group Empowerment Psychoeducation (GEP) (Figure 1). The ACT model of psychological flexibility in the center of this diagram is adapted from Hayes et al [16] and the GEP model is based on the CHAMP intervention research project previously conducted by authors Li, Fung, and Wong [17-19].

ACT is a mindfulness-based cognitive behavioral intervention that has empirical support for its use in common mental disorders including mood and anxiety disorders, and psychosis [20-24]. ACT consists of six processes to increase psychological flexibility: defusion (observing thoughts as thoughts), acceptance (opening up to experiencing thoughts and feelings), contact with the present moment (attending to the present mindfully), self-as-context (being in touch with the “observer self” and increasing perspective-taking skills), values (being clear about what matters), and committed action (developing consistent patterns of behaviors based on one's chosen values). As ACT and the practice of mindfulness are congruent with Asian traditional self-care practices and Eastern philosophy, it is especially suitable for the Chinese population. We have employed ACT clinically in the Canadian Chinese population with significant improvements in depressive symptoms as well as general functioning [25]. We have also successfully adapted it for nonclinical populations to reduce judgmental thoughts related to the stigma against HIV and mental illness in Chinese and other ethnoracial populations in Canada [26,27].

The effectiveness of psychoeducation for stigma reduction and mental distress is well established [28-30]. The GEP component of our intervention is our refined model of psychoeducation underpinned by the principles of social justice and equity; empathy and compassion; interdependence; and collective empowerment. It aims to help participants improve their capacity for self-care and stress management, develop a critical understanding of health equity, and increase their readiness to proactively participate in mental health promotion activities. The knowledge-based content includes the following: holistic understanding of mental health; common signs, symptoms, and syndromes of mental illness; and current treatment approaches and local resources. It is supported by evidence from Canadian intervention studies with Chinese and other ethnoracial immigrant communities [31,32]. The delivery utilizes four empowerment processes: critical reflection, critical dialogue, collaborative learning, and experiential learning.

The integrated ACE-LYNX intervention will be operationalized and delivered via two complementary modalities: (1) an online self-study course named Mental Health 101(MH101) and (2) a 5-day experiential group training.

### Reach, Efficacy, Adoption, Implementation, and Maintenance (RE-AIM) Project Implementation Framework

Our implementation project will be guided by the RE-AIM framework, which provides a structure to examine the processes and outcomes of implementing a public health intervention along five dimensions: reach, efficacy, adoption, implementation, and maintenance [33]. Reach measures participation at the individual level, including the risk characteristics and percentage of people who are affected by or receive a policy or program. Efficacy assesses the positive and negative outcomes, including changes in the quality of life, participant satisfaction, behavioral changes, and other clinical or nonclinical variables [33,34]. Adoption evaluates the proportion and representativeness of the settings that uptake and employ a given program or policy [35]. Implementation refers to the degree to which the intervention is conducted as intended in real-life settings, measured at the individual and program levels [33]. Finally, maintenance assesses the extent to which innovations become part of individuals’ behavioral repertoire or system changes that are relatively stable and enduring [33].

### Project Objectives for Phase Two

Guided by the RE-AIM framework, we will (1) examine the reach of the target populations by the intervention and an integrated mental health network, (2) evaluate their effectiveness, (3) gauge support for their adoption, (4) document the contextual adaptation and evaluate the fidelity in their implementation, and (5) track the maintenance and sustainability of individual behavioral changes and changes in the system processes [33].

### Project Design: Key Stages of Implementation

**Figure 2:**
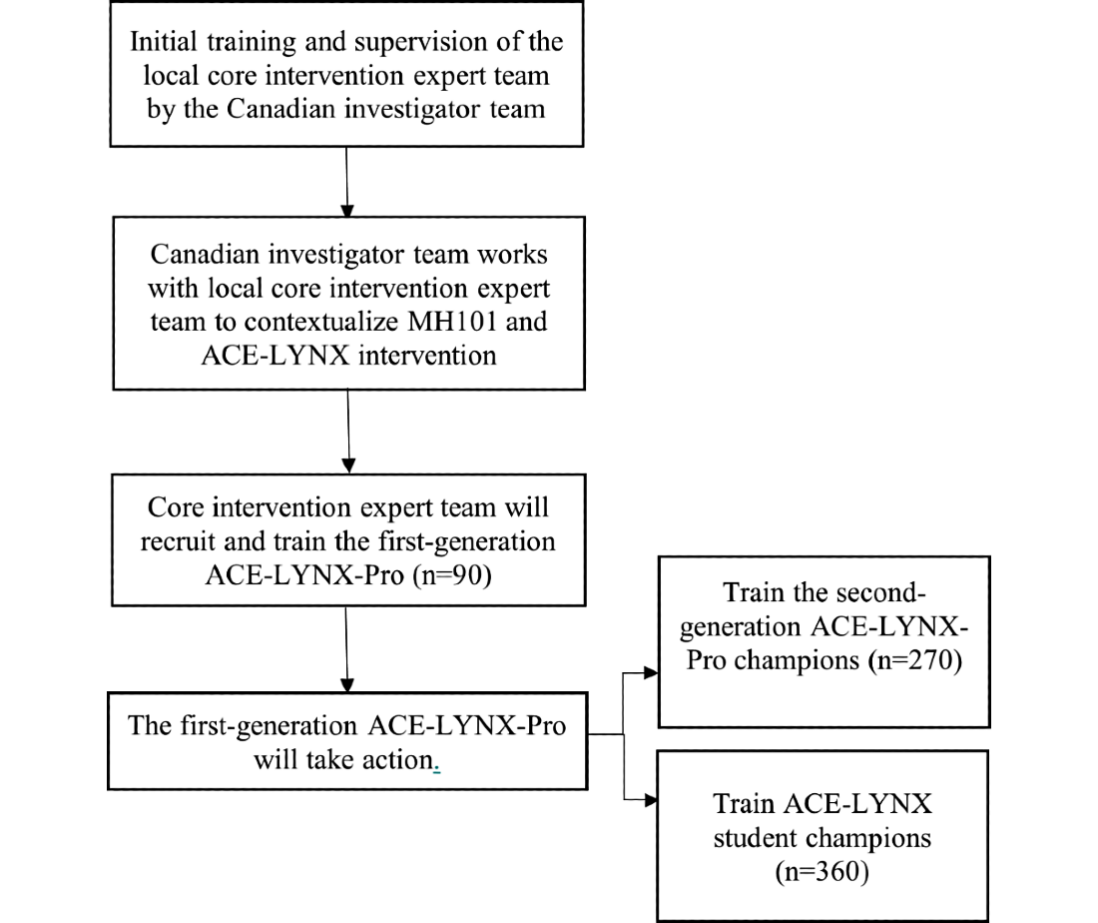
Key stages of Acceptance and Commitment to Empowerment – Linking Youth and ‘Xin’ implementation. ACE-LYNX: Acceptance and Commitment to Empowerment – Linking Youth and ‘Xin’ implementation; MH101: Mental Health 101.

Prior to the commencement of the project, research ethics approval was obtained from Canadian institutions, including Ryerson University, University of Toronto, University of Alberta, York University, and Chinese institutions including Shandong University, University of Jinan, Shandong Jianzhu University, Shandong Normal University, Shandong Women’s University, Shandong Youth University of Political Science, and Shandong Mental Health Center. A contextual assessment will be conducted with local providers, students, and other stakeholders to evaluate the priority of needs, as detailed in our protocol paper (Wong et al, under review), to facilitate implementation. The key phase two ACE-LYNX implementation steps are summarized in Figure 2 and detailed below.

#### Capacity Building: Local Core Intervention Expert Team

**Figure 3:**
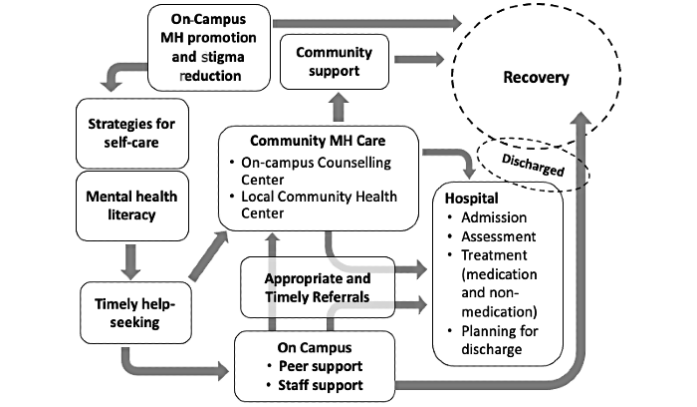
Integrated pathways of mental health care. MH: mental health.

Effective implementation of evidence-based intervention is contingent on the appropriateness of the intervention approach and specific strategies for implementing the practice in the existing systems of care [36]. From the perspective of a multilevel model of change, middle-level practice changes within organizations and teams are influenced by the policy context within the system environment as well as the attitude, knowledge, and behavior of care providers and service users at the grassroots level [36]. Therefore, we will engage and build capacity among the local health and social care professionals within the existing systems of care allocated to health promotion, treatment, and recovery services already in place, as illustrated by the integrated pathways of mental health care (Figure 3). We will use a “train-the-trainer” capacity-building model to implement and evaluate the effectiveness of the ACE-LYNX intervention designed for professionals.

A core team of 24 local interdisciplinary professionals, including psychiatrists, nurses, on-campus counselors, and social work university professors in Jinan, China will be recruited and trained by the Canadian investigator team to become the local ACE-LYNX expert team. They will serve as core intervention expert trainers for the project to deliver the intervention locally. After the initial 5-day in-person training, the core intervention expert trainers will engage in mock training and provide constructive peer feedback. The mock training will be video-recorded and reviewed by the Canadian team members for feedback via videoconferencing to ensure model fidelity. During implementation, the core intervention expert trainers will form training teams to deliver the intervention at the six universities with ongoing support from the Canadian team.

#### Contextualization of the ACE-LYNX-Pro Intervention

##### Mental Health Modules (MH101 Modules)

Key priorities and local mental health needs (Wong et al, under review) from contextual analysis will be used to locally adapt the MH101 online modules to ensure local relevance, efficacy, and acceptability. Module 1 will focus on key concepts regarding mental health and well-being drawing from positive psychology and holistic health. Module 2 will focus on factors that adversely affect mental health, including biological, psychological, sociocultural, and spiritual factors. Module 3 will introduce common mental disorders and addictions. Module 4 will discuss issues around suicide and suicide prevention, mental illness treatment and recovery, and local resources. All modules will be produced in Jinan, China, with active participation by local professors and students. The self-directed modules will be completed by all participants prior to joining the in-person group training.

#### ACE-LYNX-Pro In-Person Group Training

The ACE-LYNX-Pro group training is a 5-day intensive in-person experiential group training. During implementation, it can be flexibly scheduled as half-day (3-hour) sessions to accommodate participant needs. The first 3 days will focus on learning the core ACT and GEP processes through intense reflective and experiential group activities (see Table 1). The last 2 days will focus on the skills needed to become ACE-LYNX-Pro champions, namely delivering the training to others and taking steps toward forming a mental health network. Written informed consent will be obtained from all participants prior to the start of the training. Data from contextual analysis and inputs from the core intervention expert trainers will be used to contextualize the intervention. This includes the use of culturally appropriate translations of concepts and expressions; contextualized vignettes that reflect local experiences of stigma and mental illness; and local terms used in discussing life values.

**Table 1:** Acceptance and Commitment to Empowerment–Linking Youth and “Xin”-Pro group intervention training program.

Day	AM/PM	Session
1	AM	Introduction to ACT^a^ (theory) Health continuum (GEP^b^) Group rules/norms How to get most out of it?
	PM	Present moment I Leaves on a stream Defusion Paired singing Stigma rules and stories
2	AM	Acceptance Chair sculpture of suffering Exclusion circle
	PM	Self-as-context Le’go exercise Three things you did today Present moment II Loving kindness
3	AM	Values Cultural and personal values exercise Your legacy
	PM	Committed action Bull’s-eye and bus driver Collective bull’s-eye and committed group action project
4	AM	ACEc communication skills Group facilitation skills
	PM	Facilitating and leading ACT and experiential exercises
5	AM	Building a functional mental health network
	PM	Next steps Feedback

^a^ACT: Acceptance and Commitment Therapy.

^b^GEP: Group Empowerment Psychoeducation.

^c^ACE: Acceptance and Commitment to Empowerment

#### Selection and Eligibility of First Generation of ACE-LYNX-Pro

At each participating university, the Chinese core intervention expert team will recruit a group of 15 local psychiatric and nonpsychiatric professionals to take part in the ACE-LYNX-Pro intervention training. For each group, we aim to recruit counselors, dormitory counselors, social work professors, and psychiatrists or psychiatric nurses to promote interdisciplinary collaboration using interprofessional educational (IPE) principles [37]. After training, all participants will join a community of practice during a 3-month practicum period. They will be encouraged to post regular reflections on the impact of the intervention on their personal and professional lives. For their participation in the training program, participants will receive a certificate of completion. Effectiveness will be evaluated with psychometric scales, namely preintervention, immediately postintervention, and 3 months postintervention (see Table 2). Implementation evaluation will be guided by the RE-AIM framework (see Table 3). Participants will also be invited to join a 3-month postintervention focus group.

**Table 2:** Data collection tools.

Instrument name	Instrument description	Reliability statistics (from previous studies)
Demographics and Background Mental Health Questionnaire	This is a descriptive measure developed for the purpose of the project. Background information regarding family socio-economic status, health history, etc is collected to understand the profiles of participating individuals.	N/A^a^
Depression, Anxiety, and Stress Scale (DASS-21)	The DASS-21 is a 21-item measure of the emotional states of depression (D), anxiety (A), and stress (S) [38].	Cronbach *α*=.81(D),.89 (A), and.78 (S) [39]
Community Attitude toward the Mentally Ill (CAMI)	The CAMI is a 40-item self-reported inventory of attitudes and stigma toward mental illness [40].	Cronbach *α*=.6-.81 [41]
Mental Health Knowledge Questionnaire (MHKQ)	The MHKQ is a 20-item self-assessment to evaluate public knowledge and awareness of mental health concerns [42].	Cronbach *α*=.61 [42]
Acceptance and Action Questionnaire (AAQ-II)	The AAQ is a 7-item self-reported measure of experiential avoidance, psychological inflexibility, and cognitive fusion [43].	Cronbach *α*=.84 [43]
Bull’s-Eye	The Bull’s-Eye is an exercise to identify and measure personal values, value attainment, and persistence [44].	The test-retest reliability of the Bull’s-Eye has been high; *r*=.85 [45].
Multi-System Model of Resilience Inventory (MSMR-I)	The MSMR-I is 27-item, self-reported multidimensional measure of individual resilience capacities across intraindividual, interpersonal, and sociostructural domains [45].	Cronbach *α*=.9-.91 [46]

^a^N/A: Not applicable.

**Table 3:** Implementation evaluation using the five dimensions of the Reach, Efficacy, Adoption, Implementation, and Maintenance framework.

Dimension	ACE-LYNX-Pro/ACE-LYNX^a^ training	Integrated network
Reach	% trained and % expressing interest / estimated eligible population Comparisons: completers versus dropouts; completers versus population	# of nodes in integrated “real” and “virtual” networks tracked by SNA^b^ surveys, learning management system, and Weixin, including the # of professional champions, student champions, untrained professionals, and students
Effectiveness	Preintervention, postintervention, and 6-month follow-up: Attitude: CAMI^c^ Knowledge: MHKQ^d^ and localized questions Empowerment/MH^e^ Outcomes: DASS-21^f^, AAQ-II^g^, Bull’s-Eye, and monthly activity logs (personal, peers, university, and community)	Network structure: degree of interdisciplinary collaboration among professionals (SNA survey, learning management system); peer support and information exchange among students (Weixin); students to professional contacts (SNA survey, learning management system) Service volumes: # of new cases; diagnoses; types of services delivered between preintervention and postintervention evaluations
Adoption	Perceived feasibility, acceptability, and fit from contextual assessment (students and professional FG^h^ and advisory committees) Interest expressed for adoption by health, communities and educational organizations not involved in the project (advisory committees and iKT^i^)	Quarterly SNA survey to track programmatic, organizational, and institutional engagement by examining affiliations of champions and those that they are in contact with
Implementation	Fidelity checklist to monitor adherence to protocols and manuals: (a) IA^j^ training professionals; (b) ACE-LYNX-Pro training students; (c) ACE-LYNX-Pro training second- generation ACE-LYNX-Pro Postsession forms filled by participants Reflection forms filled by facilitators Evaluation forms filled by participants of student-driven activities	Quarterly SNA survey: density, degree centrality, centralization, and dynamic changes over time Visualization with sociograms Identification of “hubs” and “isolates”
Maintenance and sustainability	Propagation of ACE-LYNX-Pro beyond the second generation Online qualitative survey at 6 months Uptake of ACE-LYNX-Pro training model by other organizations and other educational/health institutions through iKT	Interdisciplinary consultation and support tracked through learning management system beyond 6-month practicum period Quarterly SNA survey-document longitudinal activities Dissemination of “MH101” and other mental health promotional messages through Weixin to untrained professionals and students Monthly activity logs: activities / initiatives that sustain beyond the project (eg, starting new groups, websites, etc) Uptake of network model by other educational/health setting through iKT

^a^ACE-LYNX: Acceptance and Commitment to Empowerment–Linking Youth and “Xin” implementation.

^b^SNA: Social Network Analysis

^c^CAMI: Community Attitude toward the Mentally Ill

^d^MHKQ: Mental Health Knowledge Questionnaire

^e^MH: mental health

^f^DASS21: Depression, Anxiety, and Stress Scale

^g^AAQ-II: Acceptance and Action Questionnaire

^h^FG: focus group

^i^iKT: integrative knowledge translation

^j^IA: intervention assistant

#### Recruitment and Eligibility of ACE-LYNX-Pro Champions

Over 3 months, the first-generation ACE-LYNX-Pro champions will (1) deliver the ACE-LYNX intervention (student version) to university students; (2) train interdisciplinary professionals at their university to become “second-generation” ACE-LYNX-Pro champions and provide ongoing mentorship; and (3) function as part of an integrated network to increase university students’ access to mental health care.

##### Training University Students to Become ACE-LYNX Student Champions

At each university, the 15 trained ACE-LYNX-Pro champions will form 3 5-member teams. Each team will plan, recruit, and engage a group of 20 university students to take part in the ACE-LYNX intervention (student version), aimed at reducing stigma, increasing mental health literacy, and transforming the students to become ACE-LYNX student champions. For inclusion in the intervention, students should be actively enrolled in a Linking Hearts participating university, be aged 18-24 years, be interested in promoting the health and well-being of fellow students, and be committed to connecting with fellow students on campus.

Guided by the local contextual assessment at each university, the different teams may organize gender-specific or mixed-gender training groups. The student version will consist of the online MH101 modules and four face-to-face training sessions (3.5 hours per session, 14 hours in total). The student champions will add to the informal integrated network that facilitates and promotes student access to mental health care. The trained student champions will also attend two half-day workshops that help build their capacity to promote mental health among their peers (eg, leading group sessions on mindfulness, stigma reduction, and stress management workshops). The outcome measures among student participants will be evaluated preintervention, immediately postintervention, and 3 months postintervention, whereas implementation evaluations will be undertaken concurrently (see Table 2 and 3).

Each ACE-LYNX-Pro team will also recruit a group of 15 interdisciplinary professionals from their university to undergo training, including the online modules and group training, to become second-generation ACE-LYNX-Pro champions. The outcome measures (see Table 2) will be evaluated preintervention, immediately postintervention, and 3 months postintervention. The second-generation champions will also join the integrated network to facilitate and promote student access to mental health care.

#### Forming an Integrated Mental Health Network

All trained professionals and student champions will form formal and informal community mental health networks. Professional champions will be encouraged to increase interdisciplinary collaboration, facilitate student mental health care pathways, and develop integrated programs and services. Student champions will be encouraged to carry out student-led mental health initiatives and provide peer support on campus. The integrated collaborative practice will be supported by ongoing supervision and support through a learning management system and social media platforms. The ACE-LYNX implementation stages and processes are summarized in Figure 2.

### Implementation Evaluation of ACE-LYNX

The RE-AIM framework will be used at two levels: ACE-LYNX training for professionals and students and the functioning of an integrated mental health network (see evaluation activities summarized in Table 3 and outline of project timelines in Table 4).

The reach of the ACE-LYNX training will be the proportion of professionals and students successfully trained compared to their corresponding eligible populations. The total number of eligible participants will be obtained based on the information from the advisory committees. We will examine this information for any significant differences in the demographics and other baseline variables between completers and noncompleters, and between the completers and student population data at each university. The reach of the integrated mental health care network will be determined by the parameters of the network, including the total number of trained and untrained professionals, and students who constitute and form part of the network.

**Table 4:** Overview of project activities over 5 years.

Year	Core project activities
1	Team infrastructure; capacity building: research and intervention training; training resource development
2	Contextual analysis of students and professionals; adaptation and production of online modules (MH101^a^); training, practice, and mentorship; outreach; iKT^b^ forum
3	Recruitment; first-generation ACE-LYNX-Pro intervention @ six universities; data collection and analysis; iKT seminars and forum
4	Recruitment; second-generation ACE-LYNX-Pro and ACE-LYNX^c^ student intervention @ six universities; data collection and analysis; iKT seminars and forums
5	Student support network building and student-led initiatives; final data analysis; iKT seminars, forum, and conferences

^a^MH101: Mental Health 101.

^b^iKT: integrative knowledge translation.

^c^ACE-LYNX: Acceptance and Commitment to Empowerment–Linking Youth and “Xin” implementation.

The effectiveness of the intervention will be evaluated with psychometric scales quantitatively preintervention, immediately postintervention, and 3 months postintervention, including mental illness stigma, empowerment, psychological flexibility, and other psychological self-reports (see Table 2). Behavioral outcomes will be measured using the following longitudinal data: (a) collaboration across disciplines to form networked pathways as measured by the number of connected nodes and frequency of contact using Social Network Analysis (SNA) [47]; and (b) monthly activity logs of mental health promotion and stigma-reduction activities submitted online by ACE-LYNX-Pro and student champions. Participants will also be engaged in focus groups at 3 months postintervention to identify the facilitators and barriers to apply ACE-LYNX.

The effectiveness of the functional mental health care networks in improving access to care among students will be examined considering the (1) parameters of the network structure using SNA, including the degree of interdisciplinary collaboration among professionals; the degree of peer support and information exchange among students; and the degree of student-to-professional contacts; and (2) changes in service volumes, including the number of new cases, diagnoses, and types of services used at each university counseling center between the preintervention and postintervention evaluations.

Adoption will be assessed in terms of the perceived feasibility, acceptability, and fit, as reflected by the interest and actual uptake of the ACE-LYNX intervention by the participating universities (eg, student groups and departmental initiatives) and by other nonparticipating healthcare, educational, or community organizations. This will be tracked during dissemination through iKT strategies (manuscript in preparation).

Adoption of the integrated network at the organization level will be examined by the parameters of SNA, examining the numbers and types of programs, organizations, and institutions that are affiliated with the individuals participating in and reached by the network.

Implementation fidelity checklists will be used to evaluate the quality and adherence to the ACE-LYNX model by the core intervention expert team and by the first-generation professional champions. Intervention facilitators will complete a reflection form after each session to document implementation issues and group dynamics. Participants will be asked to complete a postsession feedback form after each training session to assess their satisfaction, knowledge gain, and confidence with specific skills.

SNA will be applied to evaluate the implementation of the integrated networks, including how the relationships between and among students and service providers shape individual practices related to accessing health services and information [48]. Each university will be defined as an integrated mental health care network. The structure and function of the network will be examined, including density, degree centrality, and centralization, and their changes over time. Sociograms will be produced to examine the structure of the networks and shared with the regional advisory committees to identify potential mental health promotion “hubs” of highly connected individuals or groups that can serve as links to others in the university community for optimal knowledge dissemination.

The maintenance and sustainability of the intervention and network will be measured at the individual and organizational levels over the follow-up period, especially with a focus on activities that can sustain beyond the project and uptake by stakeholders not directly involved in the study. This includes activities captured longitudinally by monthly activity logs, SNA surveys, the learning management system, and social media platforms. In addition, an online qualitative survey will be conducted 3 months postintervention with participants and stakeholders to evaluate the intervention’s acceptability, appropriateness, and feasibility; desire for widescale adoption; and personal, professional, and community impacts of ACE-LYNX.

### Data Analysis

Quantitative data will be aggregated and descriptive. Preintervention and postintervention efficacy, and inferential analyzes will be conducted using the Statistical Package for Social Sciences (SPSS version 27.0, IBM Corp.). Trend analyses, mediation, moderation, and hierarchical modeling of data to examine the intervention mechanisms of change will be conducted using the Laavan (version 0.6-9) and Psych packages (version 2.1.6) (R-Studio). Qualitative data will be transcribed. N-Vivo will be used for data management and extraction of thematic clusters using inductive and deductive approaches.

## Results

We will directly train 90 ACE-LYNX-Pro champions (first generation), who will further train 15 professionals and 20 university students at each of the 6 participating universities. The intervention is expected to directly benefit the participants, and all the champions (n=720) will contribute to the cause by forming a mental health network and engaging in mental health promotion activities, including student-led initiatives, and peer support and professional-led efforts to increase collaborative care and facilitated care pathways.

## Discussion

The current paper outlines the key steps taken to contextualize and implement a multipronged intervention program to promote student mental health through cross-national collaboration. Based on our previous work, we anticipate that the impact of our intervention will reach 11,000 to 18,000 students through the train-the-trainer model, communities of support, and outreach activities on campus and via social media. Systemic and policy impacts are also expected through iKT strategies (paper under preparation).

The following are the unique and important elements that can substantively contribute to mental health promotion work locally and internationally: the train-the-trainer model; integrated models of individual well-being (ACT) and collective empowerment (GEP), sociocultural adaptation process; emphasis on interdisciplinary and professional–student collaboration; use of the RE-AIM framework to guide evaluation; and the examination of direct intervention impacts as well as the functioning of an emergent mental health network. The ACE-LYNX program will add to a growing body of literature in IS that identifies the strategies, challenges, and solutions for implementing evidence-based interventions to improve community mental well-being and drive system changes from the ground up.

### Declarations

#### Ethics Approval and Consent to Participate

The study protocols of the current implementation project have been approved by the research ethics boards of all participating institutions in Canada (Ryerson University 2018-455; University of Toronto 37724; York University e2019-162; University of Alberta Pro00089364) and in China (Shandong University; Jinan University; Shandong Jianzhu University; Shandong Mental Health Center; Shandong Normal University; Shandong Women's University, and Shandong Youth University of Political Sciences). All participants will provide written informed consent before participating in the study.

#### Consent for Publication

The informed consent above includes consent for the recording and publication of anonymized information shared during individual participations.

#### Availability of Data and Materials

The data from this IS study will be made available upon study completion within the extent permitted and outlined by the data sharing agreement among the partnering institutions.

## Abbreviations

ACE-LYNX: Acceptance and Commitment to Empowerment–Linking Youth and “Xin” (hearts)
ACT: Acceptance and Commitment Therapy
GEP: Group Empowerment Psychoeducation
iKT: integrative knowledge translation
IPE: interprofessional education
IS: implementation science
RE-AIM: Reach, Efficacy, Adoption, Implementation, and Maintenance
SNA: Social Network Analysis
SPSS: Statistical Package for Social Sciences
